# Characterization and Salt Response in Recurrent Halotolerant *Exiguobacterium* sp. SH31 Isolated From Sediments of Salar de Huasco, Chilean Altiplano

**DOI:** 10.3389/fmicb.2018.02228

**Published:** 2018-09-20

**Authors:** Francisco Remonsellez, Juan Castro-Severyn, Coral Pardo-Esté, Pablo Aguilar, Jonathan Fortt, Cesar Salinas, Sergio Barahona, Joice León, Bárbara Fuentes, Carlos Areche, Klaudia L. Hernández, Daniel Aguayo, Claudia P. Saavedra

**Affiliations:** ^1^Departamento de Ingeniería Química, Facultad de Ingeniería y Ciencias Geológicas, Universidad Católica del Norte, Antofagasta, Chile; ^2^Centro de Investigación Tecnológica del Agua en el Desierto (CEITSAZA), Universidad Católica del Norte, Antofagasta, Chile; ^3^Laboratorio de Microbiología Molecular, Departamento de Ciencias Biológicas, Facultad de Ciencias de la Vida, Universidad Andrés Bello, Santiago, Chile; ^4^Lake and Glacier Ecology Research Group, Institute of Ecology, University of Innsbruck, Innsbruck, Austria; ^5^Departamento de Química, Facultad de Ciencias, Universidad de Chile, Santiago, Chile; ^6^Centro de Investigación Marina Quintay, Facultad de Ciencias de la Vida, Universidad Andrés Bello, Santiago, Chile; ^7^Instituto de Ciencias Marinas y Limnológicas, Universidad Austral de Chile, Valdivia, Chile; ^8^Center for Bioinformatics and Integrative Biology, Departamento de Ciencias Biológicas, Facultad de Ciencias de la Vida, Universidad Andrés Bello, Santiago, Chile

**Keywords:** halotolerant, Chilean Altiplano, *Exiguobacterium*, extremophile, environmental pressure

## Abstract

Poly-extremophiles microorganisms have the capacity to inhabit hostile environments and can survive several adverse conditions that include as variations in temperature, pH, and salinity, high levels UV light and atmospheric pressure, and even the presence of toxic compounds and the formation of reactive oxygen species (ROS). A halotolerant *Exiguobacterium* strain was isolated from Salar de Huasco (Chilean Altiplano), a well-known shallow lake area with variable salinity levels, little human intervention, and extreme environmental conditions, which makes it ideal for the study of resistant mechanisms and the evolution of adaptations. This bacterial genus has not been extensively studied, although its cosmopolitan location indicates that it has high levels of plasticity and adaptive capacity. However, to date, there are no studies regarding the tolerance and resistance to salinity and osmotic pressure. We set out to characterize the *Exiguobacterium* sp. SH31 strain and describe its phenotypical and genotypical response to osmotic stress. In this context, as a first step to characterize the response to the SH31 strain to salinity and to establish the bases for a molecular study, we proposed to compare its response under three salt conditions (0, 25, and 50 g/l NaCl). Using different physiology, genomic, and transcriptomic approaches, we determined that the bacterium is able to grow properly in a NaCl concentration of up to 50 g/l; however, the best growth rate was observed at 25 g/l. Although the presence of flagella is not affected by salinity, motility was diminished at 25 g/l NaCl and abolished at 50 g/l. Biofilm formation was induced proportionally with increases in salinity, which was expected. These phenotypic results correlated with the expression of related genes: *fliG* and *fliS* Motility); *opuBA* and *putP* (transport); *glnA, proC, gltA*, and *gbsA* (compatible solutes); *ywqC, bdlA, luxS* y *pgaC* (biofilm and stress response); and therefore, we conclude that this strain effectively modifies gene expression and physiology in a differential manner when faced with different concentrations of NaCl and these modifications aid survival.

## Introduction

In the northern region of Chile, is located the oldest and most arid non-polar environment on Earth, the Atacama Desert (Bull et al., 2016), characterized for having soils deemed too extreme for life (Navarro-González et al., 2003). However, it comprises a wide range of ecological niches and harbors microbial diversity recently described, although culture and taxonomic identification has not been achieved (Crits-Christoph et al., 2013; Bull et al., 2016). Apart from the hyperarid and extreme hyperarid environments at the core of the desert, the region comprises the Andean plateau, the Altiplano, in this area high elevation and lower total ozone column (TOC) levels brings as a consequence high UV radiation (Bull et al., 2016; Cordero et al., 2016). Moreover, water bodies found in the Altiplano show different chemical compositions, evaporation rates, temperatures depths, among others. These variable factors trigger changes in the community structure that inhabits these lakes (Márquez-García et al., 2009; Dorador et al., 2013; Cordero et al., 2014). Reports on the microbiology composition from these environments have increased during the last decade, which reflects the widening interests in both fundamental and applied topics (Bull et al., 2016).

Salar de Huasco is a high altitude (3800 m.a.s.l) saline wetland composed by a complex system composed of various ground sources, streams, and shallow permanent and non-permanent ponds (Dorador et al., 2008b, 2010). This wetland presents poly-extreme environmental conditions that include a broad range of salinity ranging from freshwater to saturated salt waters, negative water balance, large daily thermal amplitude (-10 to + 25°C), low atmospheric pressure, and one of the highest solar radiations registered in the world (over 1000 Wm^-2^; Risacher et al., 2003; Hernández et al., 2016; Molina et al., 2016). There are several reports of the composition of microbial communities including *Proteobacteria, Cyanobacteria*, ammonia-oxidizing bacteria, *Bacteroidetes, Verrumicrobia, Firmicutes*, and *Archaea* (Dorador et al., 2008a,b, 2009, 2010, 2013). Bacterial diversity and active community belonging to most of these groups have been corroborated using pyrosequencing approaches (Aguilar et al., 2016; Molina et al., 2016). Moreover, it was demonstrated that this system presents a large percentage of unclassified sequences suggesting the existence of large, undiscovered bacterial diversity (Aguilar et al., 2016), and the diversity and structure of active bacterial community is extremely dynamic throughout the day subjected to nutrient recycling (Molina et al., 2016). In this environment, there have been isolated microorganism with relevant traits that have been recently studied with the “omics” approaches that include high-throughput quantitative proteomics and comparative genomics analysis, for example, the survival of *Rhodocater* sp. has been studied under extreme UV radiation and other environmental stress conditions (Pérez et al., 2017, 2018), and the phylogenetic placement of *Exiguobacterium* sp. SH31 and its possible genetic determinants required for the response to stress (Castro-Severyn et al., 2017), respectively. Therefore, Salar de Huasco represents a reservoir of model microorganisms to study response mechanisms to a wide range of stress factors.

Halotolerant bacteria are those capable of growing in the absence as well as in the presence of relatively high salt concentrations (if growth extends above 2.5 M are known as extremely halotolerant; Kushner, 1978). Overtime there has been isolated and taxonomic characterized a large number of moderately halophilic bacteria (Oren, 2008), and many of them belong to phyla *Cyanobacteria, Proteobacteria, Firmicutes, Actinobacteria, Spirochaetes*, and *Bacteroidetes* (Ventosa et al., 1998; Oren, 2008). One of the most important strategies used by halotolerant microorganisms to thrive in high salinity is the transport and/or biosynthesis of organic solutes (Imhoff, 1986; Ventosa et al., 1998; Kempf and Bremer, 1998; Müller and Oren, 2003; Roberts, 2005; Ma et al., 2010). Interestingly, most halophilic bacteria maintain intracellular cocktails of different compatible solutes (Ventosa et al., 1998; Roberts, 2005), and these molecules can also protect microorganisms against other stresses, namely, dehydration, heat, desiccation, freezing and UV radiation (Kempf and Bremer, 1998; Welsh, 2000; Lebre et al., 2017; Pérez et al., 2017). However, in some Gram-positive bacteria, the response to different salt concentrations may affect fatty acids in the membrane lipids, stress response, protein quality control, endospore germination, chemotaxis, and motility (Ventosa et al., 1998; Steil et al., 2003; Feng et al., 2007; den Besten et al., 2009; Hahne et al., 2010; Lopalco et al., 2013).

The versatile genus *Exiguobacterium* is a highly diversified group of pigmented Gram-positive bacteria with variable morphologies, ranging from small rods to cocci, and have adapted to a large variety of habitats (Collins et al., 1983; Vishnivetskaya et al., 2009; Kasana and Pandey, 2018). To date, a large number of *Exiguobacterium* strains have been isolated from different habitats with the highlighted relevance of its ability to thrive in a wide temperature range that pose possible biotechnological applications (Vishnivetskaya and Kathariou, 2005; Vishnivetskaya et al., 2007, 2009; Kasana and Pandey, 2018). This genus has been divided into two major groups based on taxonomic and phylogenetic analysis of the genus (using 16S rRNA gene sequences), group I comprises strains isolated from cold environments and group II includes strains from alkaline marine environments and hot springs (Vishnivetskaya et al., 2009). Furthermore, a recent comparative genomic analysis of 34 available *Exiguobacterium* genomes proposed that there are six clusters (two within group I and four within group II) grouped by an ANI cut-off value of 75%, this value was necessary to achieve groups formation, due to the high degree of divergence presented by this genus. Moreover, the same work revealed differences between the two groups with respect to the presence of stress-response genes, which were observed on pigment biosynthesis, osmo-adaptation, oxidative stress, capsule biosynthesis, DNA repair, and cold shock genes, using bidirectional BLAST-based approach. The strains isolated from each environment present a particular gene repertoire that correlates to those extreme conditions (Castro-Severyn et al., 2017).

As a consequence of the environments in which this microorganism is able to live, it has developed several traits of biotechnological interest, namely, reduce mercury and chrome, and arseneate to arsenite, neutralize alkaline waste water, and remove pesticide additionally, some strains are able to function on variable ranges of temperatures, pH, and salt concentrations. Numerous strains of the genus *Exiguobacterium* have been isolated from saline environments that include saline soils, salterns, wetlands, high-altitude lakes, among others, showing its plasticity and adaptation capability (Rebollar et al., 2012; Ordoñez et al., 2013; Paul and Lade, 2014; Castro-Severyn et al., 2017; Murthy and Gayathri, 2017); however, the response from these bacteria to salinity has been poorly studied. Most of the findings show ranges of tolerance to NaCl in the genus *Exiguobacterium* (between 0–20 g/l) have focused on the description of type-strains and genomes announcement (**Supplementary Table S1**). On the other hand, some salt tolerant *Exiguobacterium* strains have showed possible applications in bio-removal of hexavalent chromium from water (Okeke, 2008), reduction of dissolved organics presents in tannery saline wastewater (Sivaprakasam et al., 2008), treatment of azo dye wastewater (Tan et al., 2009), and plant growth-promoting in salt stressed soils for cultivation (Bharti et al., 2013).

We aimed to understand the salt response mechanisms in the *Exiguobacterium* genus, a group of bacteria with relevant characteristics and high plasticity that promotes it as an ideal subject to evaluate adaptation strategies that allows it to survive under extreme conditions, hence we used as a model the strain SH31 isolated from sediments from poly-extreme Salar de Huasco. The genome from this strain was recently sequenced, and several genetic determinants required for the response to stress were identified (Castro-Severyn et al., 2017). As this strain possesses several adaptation traits at the genome level, we set up to characterize the recently discovered SH31 strain and to describe its physiological response or adaptation against saline stress. For this, we determined the recurrence of *Exiguobacterium* strains in water and sediment of three sites with different salinity levels. Additionally, we phenotypically characterized the strain SH31 (shape, presence of flagella, pigmentation), and determined the effect of NaCl concentration on its growth, motility, fatty acid composition, and biofilm formation. Finally, we searched compatible solutes synthesis and transport genes in the strain SH31 and sequenced members of *Exiguobacterium* genus, and we determined the transcriptional expression of genes of interest that are related to osmoprotection.

## Materials and Methods

### Site Description and Sample Collection

During January 2011, we collected water and sediment samples from three sites in the Salar de Huasco (3,800 m altitude). The salar shows high spatial heterogeneity, represented by shallow permanent and non-permanent lagoons, streams, bofedales (peatlands), and salt crusts (Dorador et al., 2008b). The sampling sites (H3, H4, and H6) were selected because they showed different levels of salinity (**Table 1**). Salinity and conductivity were recorded with a Hanna HI 98192 meter, and pH with a Hanna HI 8314 meter.

**Table 1:** Location and characteristics of sampling sites at Salar de Huasco.

Site	Location	Salinity	Conductivity	pH
H3	S 20° 16′ 59.2″ W 068° 53′ 17.2″	0.3%	623 μS/cm	8.60
H4	S 20° 17′ 41.6″ W 068° 53′ 17.3″	12.3%	20,600 μS/cm	8.81
H6	S 20° 19′ 42.3″ W 068° 51′ 10.1″	1.2%	2,300 μS/cm	8.60


### Enrichment and Isolation of Halophilic Bacteria

Samples of water and sediment were collected at three sites of Salar de Huasco. Upon collection, samples were inoculated into YP culture medium (2 g/l yeast extract, 5 g/l Peptone and different salt concentrations [0, 25, 50, and 100 g/l NaCl]) and incubated at 25°C for 24 h. Enrichments were then plated in marine broth (prepared following the manufacturer’s instructions – Difco) and YP medium (both including 12 g/l of agar). The plates were incubated at 25°C until the appearance of colonies. All colonies (50 in total) were re-isolated into YP culture media and the salt tolerances (within a range of 0–150 g/l) were tested.

### Molecular Identification and Phylogenetic Analysis

DNA from isolated halophilic bacteria was extracted using Ultra Clean Soil DNA Isolation Kit (MoBio Lab., Inc.). For PCR-amplification of bacterial 16S rRNA genes, 27F and 1542R primers (Stackebrandt et al., 1993) were used. Each PCR reaction contained 5x PCR-buffer with 1.7 mM MgCl_2_ (Roche), 2 mM dNTP mixture (Gibco), 0.8 μM of each primer, 1.25 U Taq polymerase (PROMEGA), 10–50 ng template DNA, and MiliQ water to a final volume of 25 μL. PCR reactions were performed using the following conditions: initial denaturing step of 5 min at 94°C, followed by 30 cycles of denaturing at 94°C for 45 s, annealing at 40°C for 45 s, elongation at 72°C for 1.5 min, and a final elongation step at 72°C for 5 min. 16S rRNA sequences of phylotypes were compared with GENBANK using a BLAST search (accession numbers are shown in **Table 2**; Altschul et al., 1990). A total of 64 sequences including the isolated and closest relatives were aligned using MUSCLE (Edgar, 2004) and a phylogenetic tree was constructed using MEGA6 (Tamura et al., 2013) with the maximum likelihood method based on general time reversible (GTR) model (Nei and Kumar, 2000). A total of 1,000 iterations were used.

**Table 2:** Halophilic bacterial phylotypes isolated from water and sediment samples of Salar de Huasco.

Phylotype	Sample	Site	Isolates	AN	First hit Blastn	Identity	NaCl tolerance
1	Water	H6	**1a**	KU696292	*Shewanella baltica* strain 63	99%	0–25 g/l
2	Water	H6	**2a**	KU696293	*Pseudoalteromonas* sp. BSs20043	98%	25–50 g/l
3	Water	H3, H6	3a, 6a, **7a**, 21a	KU696289	*Halomonas* sp. B01	99%	25–100 g/l
4	Water	H3, H6	4a, 8a, 19a, **22a**	KU696291	*Aeromonas* sp. Z2_S_TSA18	99%	0–50 g/l
5	Water	H3, H4, H6	**5a**, 13a, 15a, 17a	KU696287	*Exiguobacterium* sp. AC-SC-C2	99%	0–50 g/l
6	Water	H4	**9a**, 10a	KU696294	*Pseudoalteromonas aliena* strain EH1	99%	25–100 g/l
7	Water	H4	11a, 12a,**14a**	KU696288	*Pseudomonas guineae* strain LMG 24016	99%	0–25 g/l
8	Water	H3	**18a**	KU696290	*Erwinia aphidicola* strain LMG 24877T	99%	0–50 g/l
9	Water	H3	**20a**	KU696286	*Exiguobacterium undae* strain GLPB9	99%	0–50 g/l
10	Sediment	H3, H4	**23a**, 41a, 44a,46a	KU696302	*Halomonas neptunia* strain MAT-17	98%	25–100 g/l
11	Sediment	H3, H4	**24a**, 43a	KU696303	Uncultured *Pseudoalteromonas* sp. Clone C146500413	99%	25–50 g/l
12	Sediment	H4	**25a**	KU696301	*Halomonas ventosae* strain XJSL6-9	99%	25–100 g/l
13	Sediment	H4	**27a**	KU696308	*Staphylococcus warneri* strain 41cp	100%	0–100 g/l
14	Sediment	H3, H4, H6	28a, 29a, **31a**, 34a, 38a, 45a, 47a, 48a, 49a, 50a	KU696296	*Exiguobacterium* sp. AC-SC-C2	99%	0–50 g/l
15	Sediment	H4	**30a**	KU696298	*Marinobacter excellens* strain KMM 3809	99%	5–100 g/l
16	Sediment	H6	**32a**	KU696300	*Marinobacter persicus* strain M9B	98%	25–100 g/l
17	Sediment	H6	33a, **39a**	KU696306	*Vibrio metschnikovii* strain NB9	99%	0–50 g/l
18	Sediment	H3, H4	**34a**, 49a	KU696297	*Bacillus methylotrophicus* strain CBMB205	99%	0–100 g/l
19	Sediment	H6	**35a**	KU696295	*Halomonas* sp. GT	99%	25–100 g/l
20	Sediment	H6	**36a**	KU696307	*Salinivibrio* sp. S10B	97%	25–200 g/l
21	Sediment	H6	**37a**	KU696304	*Idiomarina loihensis* GSL 199	99%	25–50 g/l
22	Sediment	H6	**40a**	KU696305	*Enterobacter aerogenes* strain PSB28	99%	0–50 g/l
23	Sediment	H3	**42a**	KU696299	*Halomonas* sp. M45-2N	96%	25–100 g/l


*The bold numbers are the sequences deposited at GenBank. AN, accession number.*

### Electron Microscopy

Morphology and flagellum presence in the *Exiguobacterium* sp. SH31 strain was examined by transmission electron microscopy (TEM), cells were grown in three separated conditions (YP medium with 0, 25, and 50 g/l NaCl), at 25°C, until the early stationary phase. After, the cells were washed with ultra-pure water and were later suspended to an OD_600_ of 0.5 and aliquots of 10 μl were placed onto carbon-coated nickel grids. After the microorganisms settled in the grid for 10 min, the samples were dried as described previously (Remonsellez et al., 2006). Finally, a transmission electron microscope (Philips Tecnai 12), operating at 80 kV, was used to obtain images.

### Extraction of Pigment and Spectrophotometric Analysis

Cells of *Exiguobacterium* strain SH31 were grown until the early stationary phase in YP culture media with 25 g/l NaCl and were harvested by centrifugation at 7,700 *g* for 15 min. Pellets were washed with sterile distilled water and spun at 1,000 *g* for 15 min. Each pellet was suspended in 5 ml absolute methanol, subjected to vigorous vortex for 2 min, followed by a resting period of 10 min and centrifuged at 4,000 *g* for 15 min. The colored supernatant was filtered through Whatman no.1 filter paper. The absorption spectrum of the pigment extract was measured within wavelengths of 270–660 nm in a UV–visible spectrophotometer UV 1800 (Shimadzu).

### Effect of Salinity on Phenotypic Properties

The metabolic capacity and utilization of organic substrates as sole carbon sources by *Exiguobacterium* sp. SH31 under different NaCl concentrations (0, 25, and 50 g/l) was tested using Biolog GP2 MicroPlates (Microlog Systems) according to the manufacturer’s instructions. YP medium plus the different NaCl conditions was used instead of Biolog Universal Growth agar medium after bacterial culture with the same conditions as before. Cell suspensions were prepared in the inoculating fluid (IF GN/GP). The inoculated plates were incubated for 24 h and the results were read (OD_590_) with a multimode plate reader (Tecan Infinite M200 Pro). The analysis was carried out as recommended by Garland (1997).

### Effect of Salinity on Growth

The *Exiguobacterium* strain SH31 was grown in YP culture media with 0, 25, 50, and 75 g/l NaCl. Cells were incubated at 25°C and orbital agitation at 120 rpm for 75 h was performed. These experiments were performed with previously adapted cells to their respective salt concentrations. Growth was monitored by measuring OD_600_ in a UV–visible spectrophotometer UV 1800 (Shimadzu).

### Effect of Salinity on Fatty Acid Composition

To determine the effect of NaCl on fatty acid composition of the *Exiguobacterium* strain was grown at 25°C in YP medium with the three different NaCl concentrations (0, 25, and 50 g/l) separately, until the early stationary phase. Cells from 0.5 l of each culture were lyophilized and mixed with methanol:hydrochloric acid:chloroform (10:1:1 v/v) for transesterification, as previously described (Miller and Berger, 1985). The fatty acids were identified as methyl esters using gas chromatography-mass spectrometry (Focus Clarus 680, Perkin Elmer) coupled with a mass spectrometer (Clarus SQ 8T model, Perkin Elmer) equipped with a DBP-1 capillary column (30 m × 0.2 mm, i.d. × 0.33 μm). Helium was used as a carrier gas at a flow rate of 1 ml/min. Oven temperature was initially kept at 150°C for 10 min, ramped at 4°C/min to 300°C, and held for 5 min. Spectra were recorded in full scan (from 50 to 500 *m/z*). The esterified fatty acids were identified by comparing the mass spectra with the NIST MS 2.O library data.

### Effect of Salinity on Motility

Swimming motility assays were done as previously described with minor modifications (Roeßler et al., 2000). *Exiguobacterium* strain was grown in the solid YP medium (0.3% of agar-Difco) and the petri dish was inoculated with a drop over the agar in the center. Assays were performed in the absence (0 g/l NaCl) and in the presence of NaCl (25 and 50 g/l NaCl), and the motility ratios were determined after 72 h of incubation at 25°C.

### Effect of Salinity on Biofilm Formation

Biofilm formation assays were examined on polystyrene plates using crystal violet (CV) staining (Pratt and Kolter, 1998). Assays were done as described previously with the following modifications (Ueda and Wood, 2009). Overnight cultures of the *Exiguobacterium* strain SH31 grown in YP medium with 0, 25, and 50 g/l NaCl were diluted to OD_600_ of 0.05 with fresh YP medium with their respective salt concentrations, and then 1 ml of diluted bacterial culture in quadruplicate were incubated in 48-well polystyrene plates for 50 h at 25°C. Later, the bacterial cultures were removed from wells. After wells were stained with 1 ml of 1% CV, rinsed and thoroughly dried, the CV was solubilized by the addition of 1.2 ml of ethanol–acetone (80:20). 1 ml of the solubilized samples were used to determine the OD_570_ using a UV–visible spectrophotometer UV 1800 (Shimadzu). The OD_570_ values were normalized using the cell density value (OD_600_) of overnight culture for each condition (0, 25, and 50 g/l NaCl).

### *Exiguobacterium* Genomic Dataset and Orthologous Search

All available genome sequences used in the analyses have been deposited in GenBank as of May 2017 (**Supplementary Table S2**). The resulting 42 genomes were organized into 22 described species, plus 20 not classified at the species level. All the genomes were re-annotated using a combination of *ab initio* and similarity methods as implemented in Prokka version 1.10 (Seemann, 2014) in order to even all the annotations and make them more comparable. We set out to find on the SH31 strain genome, 15 genes (**Supplementary Table S3**) reported as related to processes of bacterial osmotolerance using BLAST (Altschul et al., 1990). We downloaded protein sequences for each one of these genes from Swiss-Prot (Bairoch and Apweiler, 2000) and performed a reciprocal BLAST (tblasn) against SH31 genome, to infer its presence and homology. Following, we search on the genomes data set for the presence and copy number of these genes, using the best hits, through BLAST. A maximum *e*-value of 1E^-05^ and a query coverage filter of 85% were used to avoid partial alignments. This strategy was used to compare osmotic stress related genes through the whole data set, as well as pigment synthesis ones on the SH31 strain.

### Relative Expression of Genes Related to Osmoprotection

To determine the relative expression of genes involved in osmoprotection that are present in *Exiguobacterium* SH31: *opuBA, putP, glnA, proC, gltA, gbsA, fliG, fliS, ywqC, bdlA, luxS*, and *pgaC*, transcripts levels were quantified by qt RT-PCR. *Exiguobacterium* SH31 was grown in YP medium at 25°C with constant agitation in the three different conditions (0, 25, and 50 g/l of NaCl) until reaching an OD_600_ of 0.4. At this point, the cultures were pelleted and RNA extractions were carried out using the GeneJET RNA Purification Kit (Thermo Scientific) according to manufacturer’s instructions. RNA integrity, quality, and quantity were verified using 1% agarose electrophoresis and OD_260/280_ ratios. cDNA was synthesized using the M-MLV Reverse Transcriptase kit (Promega) and Random Primer oligonucleotides hexamers (Invitrogen^TM^). The PCR reaction was carried out as follows: 10 minutes at 95°C followed by 40 amplification cycles (95°C × 30 s, 58°C × 30 s, 72°C × 30 s), and a final step of 95°C × 15 s; 25°C × 1 s; 70°C × 15 s; and 95°C × 1 s) using primers specific for each gene (**Supplementary Table S4**). Transcript levels were quantified using the Brilliant II SYBR Green qPCR Master mix kit (Agilent Technologies) on a Stratagene Mx3000P thermal cycler. Gene expression levels were calculated according to Pfaffl (2001) using 16S rRNA gene as normalizator.

### Statistical Analysis

All the assays were performed in at least three independent experiments with three technical replicates each. One-way ANOVA with *post hoc* Tukey HSD test was used for all comparisons and a *P*-value < 0.05 was considered statistically significant. All the statistics were performed using GraphPad Prism 5.0, (Prism^®^, San Diego, CA, United States^1^).

## Results

### Diversity and Recurrence of Isolated Halophilic Bacteria

Halophilic bacteria were isolated from three study-sites that had similar pH values, but marked differences in salinity and conductivity values, which have been defined in previous works (Dorador et al., 2008a, 2010). The sampling sites from north to south include: H3: shallow lagoon with low salinity, H4: shallow hypersaline lagoon, and H6: anoxic lagoon with fluctuating water levels and high salinity (**Table 1**). A total of 50 isolates were obtained in this study, and 21 of these isolates come from water samples and 29 from sediments. All of them were classified within *Gammaproteobacteria* class and *Firmicutes* phylum (**Supplementary Figure S1**). In the water samples, we observed seven different genera that included *Aeromonas, Erwinia, Halomonas, Pseudoalteromonas, Pseudomonas*, and *Shewanella*, with only *Exiguobacterium* representing *Firmicutes*. Members of *Halomonas* and *Exiguobacterium* genera were found in all waters samples (**Table 2**). Instead, within the 11 genera detected in the sediment samples, it was observed the presence of *Enterobacter, Halomonas, Idiomarina, Marinobacter, Pseudoalteromonas, Pseudomonas, Salinivibrio*, and *Vibrio*; and *Bacillus, Exiguobacterium*, and *Staphylococcus* as part of *Firmicutes*. Although members of *Halomonas* and *Exiguobacterium* genera were also detected in all sediment samples, the latter one was the most abundant isolate (10 isolates), this may be a cause of favoring culture conditions and medium. The phylogenetic analysis of the most abundant phylotype (represented by strain SH31) showed 99% similarity in the 16S rRNA gene sequence with *Exiguobacterium* sp. AC-SC-C2 (FJ231171) and *Exiguobacterium aurantiacum* DSM 6208 (type-strain). Moreover, the salt tolerance of phylotypes showed the presence of strict halophiles and halotolerant between the isolated bacteria, in which *Exiguobacterium* strains were able to tolerate up to 50 g/l NaCl (**Table 2**). Therefore, it is notable that *Exiguobacterium* is a recurrent halotolerant cultivable bacterium in this poly-extremophilic ecosystem.

### Characterization of the Recurrent *Exiguobacterium* Strain, SH31

**FIGURE 1:**
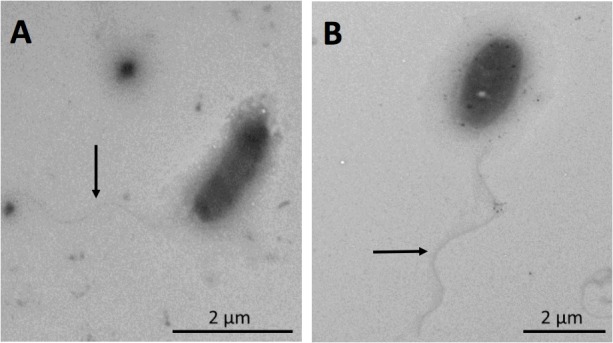
Visualization of *Exiguobacterium* strain SH31 cells grown in the presence of NaCl by transmission electron microscopy (TEM). Cells were grown to early stationary phase in 0 g/l **(A)** and 25 g/l **(B)** NaCl. Arrows indicate the presence of flagella.

The isolated strain, SH31, showed rod morphology, only one polar flagellum, which was not altered by the absence (0 g/l) or presence (25 g/l) of NaCl in the growing culture (**Figure 1**). The same phenomena were observed in the presence of 50 g/l NaCl (data not shown). On the other hand, SH31 strain colonies are regular, circular, and orange-pigmented (**Supplementary Figure S2A**). The methanolic extract of SH31 cells analyzed spectrophotometrically by scanning the absorbance within a wave length region 270–670 nm demonstrated the presence of a shoulder peak with a maximum absorbance of λ = 465 (**Supplementary Figure S2B**). Moreover, in regard of the spectrophotometric determination of SH31 pigments, the genomic analysis showed, as we expected, the presence of genes related to carotenoids biosynthesis (*crtB, crtD, crtP*, and *carC*) located in the same gene context with other genes that have been referenced as participants in processes like L-arginine and uridine monophosphate biosynthesis and others (*carH, carD, carA, pcs*, trans-aconitate 2-methyltransferase, N-glycosyltransferase, acyltransferase; **Supplementary Figure S2C**).

Phenotypic properties such as the ability of the SH31 strain to utilize or oxidize different carbon sources was tested to yield a characteristic pattern, which could constitute a metabolic fingerprint for this strain and for each condition tested. The pattern of strain SH31 presented a level of similarity of 49% with respect to *E. aurantiacum* DSM 6208 (type-strain) in the control condition (NaCl 0 g/l) and comparing the three NaCl conditions (only in SH31 strain), we find that the pattern does not turn out to be logic or viable, because as the concentration of salt in the test increases, the amount of positive reactions decreases (**Supplementary Table S5**). This phenomenon may be due to the fact that the high NaCl concentration in the tests causes some type of interference in the reaction, so it is unable to yield an appropriate result.

### Effect of Salinity on Growth, Fatty Acids, Motility, and Biofilm Production in Strain SH31

**FIGURE 2:**
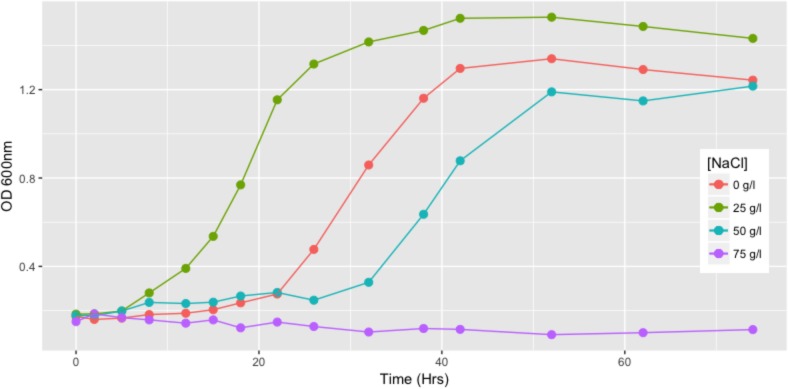
Growth of *Exiguobacterium* sp. SH31 in the presence of NaCl. Strain SH31 cells were grown in their respective growth media in absence of NaCl or supplemented with 25, 50, or 75 g/l NaCl. OD_600_ readings were recorded during 74 h. Mean values (*n* = 3) are plotted.

The growth curve of the SH31 strain reveals its ability to replicate in the presence of high salt concentrations. We found that at 25 g/l of NaCl this strain exhibits its best growth behavior, reaching an OD_600_ value of 1.4 and stationary phase earlier than the other conditions evaluated (around 24 h). SH31 is classified as halotolerant, interestingly, in the absence of NaCl; SH31 is also able to grow well, although it exhibits slower and poor growth in the 50 g/l concentration. We also found that 75 g/l completely inhibits the ability of the SH31 strain to survive and replicate itself (**Figure 2**).

The major amounts of fatty acids found in strain SH31 were iC_15:0_, C_16:0_, and C_17:0_ and other minor components listed in **Table 3**. The proportion of fatty acids of strain SH31 did not change with varying salt concentrations in the medium. Although it was previously evidenced that SH31 strain is phylogenetically related to *Exiguobacterium mexicanum* and *Exiguobacterium auriantiacum* (Castro-Severyn et al., 2017), our results regarding fatty acids patterns show differences between then (**Table 3**). However, *E. mexicanum* and *E. auriantiacum* type-strains show some resemblance, although iC_17:0_ and C_18:1_ω_9c_ are absent in *E. mexicanum* while C_16:1_ω_7c_ is absent in *E. auriantiacum* (**Table 3**). The fatty acid patterns of other members of the *Exiguobacterium* group II (*Exiguobacterium alkaliphilum* and *Exiguobacterium marinum*) also show differences with the SH31 strain (Kim et al., 2005; Kulshreshtha et al., 2013).

**Table 3:** Fatty acid composition of *Exiguobacterium* sp. SH31 grown in different NaCl concentrations and comparison with the closer type strains.

Fatty acid	*E.* sp. SH31 (This work)			*E. mexicanum DSM 16483T*	*E. auriantiacum DMS 20416T*
			
	0 g/l NaCl	25 g/l NaCl	50 g/l NaCl	(López-Cortés et al., 2006)	(Frühling et al., 2002)
*i*C_7:0_	0.09	0.23	0.28	–	–
3-(methyltio)C_3:0_	0.09	0.38	0.41	–	–
C_4:0_ (diacid)	0.57	0.73	0.68	–	–
iC_11:0_	–	–	–	1.5	2.0
iC_12:0_	–	–	–	2.1	3.0
C_12:0_	–	–	–	8.1	2.0
iC_13:0_	0.21	0.18	0.19	11.2	18.0
aiC_13:0_	–	–	–	8.3	12.0
C_13:0_	0.08	0.12	0.10	–	–
iC_14:0_	1.01	0.38	0.3	–	–
C_14:0_	3.77	2.55	2.51	6.1	3.0
iC_15:0_	62.01	65.44	65.14	1.7	4.0
C_16:1_w_7c_	–	–	–	6.5	–
C_16:1_w_9c_	0.44	0.28	0.32	–	–
C_16:1_w_11c_	–	–	–	10.3	10.0
iC_16:0_	4.19	2.52	2.72	–	–
C_16:0_	9.67	11.94	12.03	32.8	27.0
iC_17:0_	0.61	1.46	1.44	–	6.0
C_17:0_	16.69	13.38	13.12	–	–
C_18:1_w_9c_	–	–	–	–	2.0
C_18:0_	0.57	0.41	0.43	7.0	5.0


**FIGURE 3:**
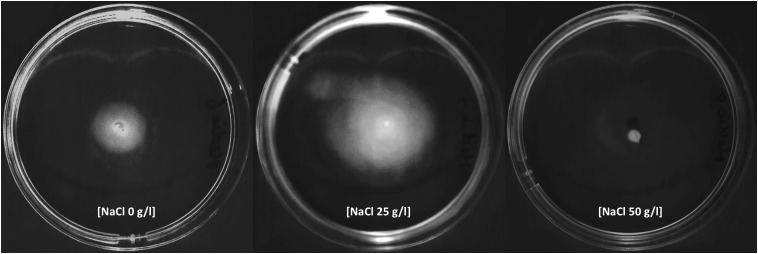
Effect of salinity in motility of *Exiguobacterium* strain SH31. The microorganism was adapted to grow with 0, 25, and 50 g/l NaCl. Cells were inoculated in their respective salts concentration in swim plates and photographed after 72 h of incubation at 25°C.

**FIGURE 4:**
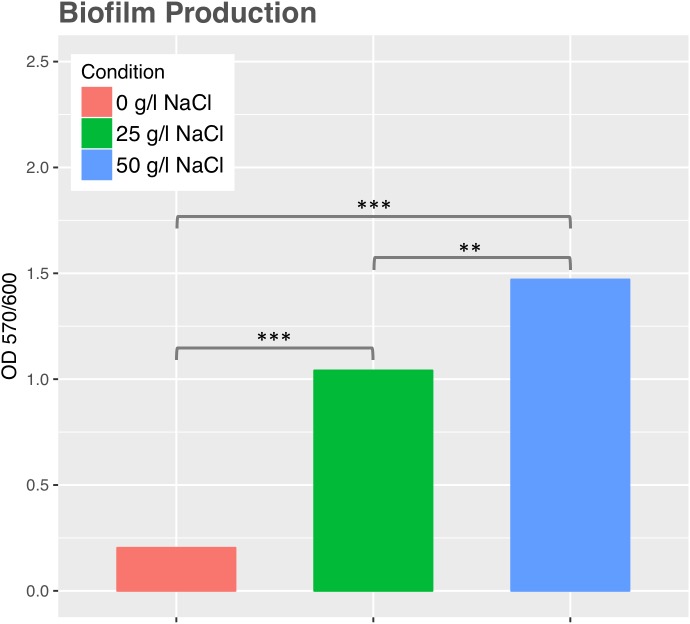
Biofilm production by *Exiguobacterium* sp. SH31 under different NaCl conditions. Data represents an average of three independent experiments with three technical replicates each (^∗^*p* < 0.05; ^∗∗^*p* < 0.01; ^∗∗∗^*p* < 0.001).

Swimming motility was observed in cultures exposed to 0 and 25 g/l NaCl and showed the highest motility ratio at 25 g/l of NaCl (**Figure 3**). However, motility was completely inhibited at 50 g/l. The highest motility was reached in the same NaCl concentration where SH31 strain exhibited its best growth (**Figure 2**). Nonetheless, at higher concentrations, it is still able to survive, and although it has flagellum (as confirmed by TEM, **Figure 1**), it is not motile, which suggests that at these salt concentrations, the cell is less active or could be under some kind of regulation by environmental cues (Chatterjee et al., 2010). On the other hand, it has been reported that several bacterial groups are able to generate extracellular compounds to establish their niche and survive. Specifically, bacteria use biofilms as a structure to resist adverse conditions. Here we find that SH31 strain increases the production of biofilm proportionally to salt concentrations in the media (**Figure 4**), this phenomenon is very common among extremophile bacteria or those that face extreme conditions, in which this structure is used as protection (Le Magrex-Debar et al., 2000; Hall-Stoodley et al., 2004; Thormann et al., 2005).

### Genomic Analysis

**FIGURE 5:**
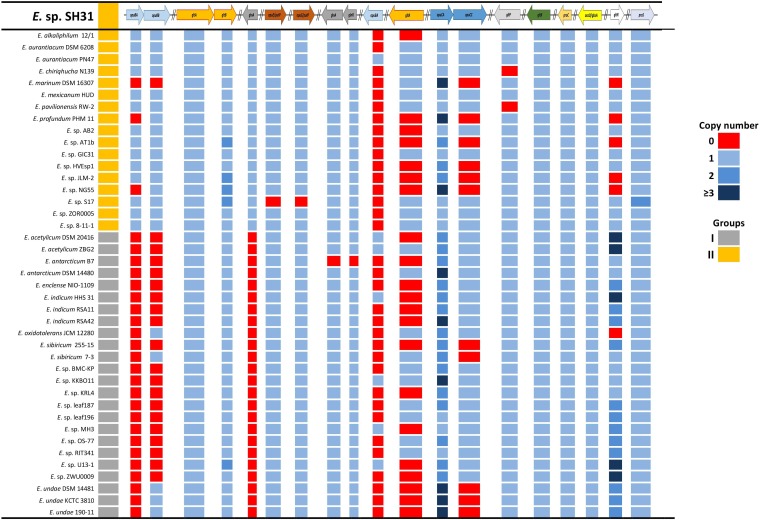
Compatible solutes synthesis and transport genes in sequenced *Exiguobacterium* strains. The first column indicates Vishnivetskaya et al. (2009) grouping, and colors indicate the copy number of genes.

To assess the genomic possibilities of the *Exiguobacterium* genus, and specifically the SH31 strain’s ability to cope with highly saline environments, we used a reciprocal BLAST approach against Swiss-Prot. The SH31 strain genome was searched for the presence of osmoprotection genes in the way of transport and compatible solutes biosynthesis. In this search, we selected 15 genes to use as queries against all 41 other *Exiguobacterium* genomes and searched for the orthologous genes and the copy number of each gene (**Figure 5**). Overall, we found that all the genomes have a good repertoire of genes related to this function. The important variation (revealed in the dataset) is related to gene copy number and, in most cases, not related to a specific group, contrary to what was previously reported on this genus for most of the arsenic resistance genes (Castro-Severyn et al., 2017). In only a few cases, we observed some kind of group pattern, specifically *opuBA, opuBB*, and *glnA* genes, which are generally absent in group I, the first two genes are part of a choline transport system and the last with a glutamine synthesis process. Other particular cases (like the *gltB* gene, which is involved in the glutamate synthesis) is absent in some group II strains and in some strains of group I have multiple copies. The copy number of these genes in each strain may be very particular or specific to its own niche, or the specific conditions that each one of the strains faces.

### Transcriptional Expression of Genes Related to Osmoprotection

**FIGURE 6:**
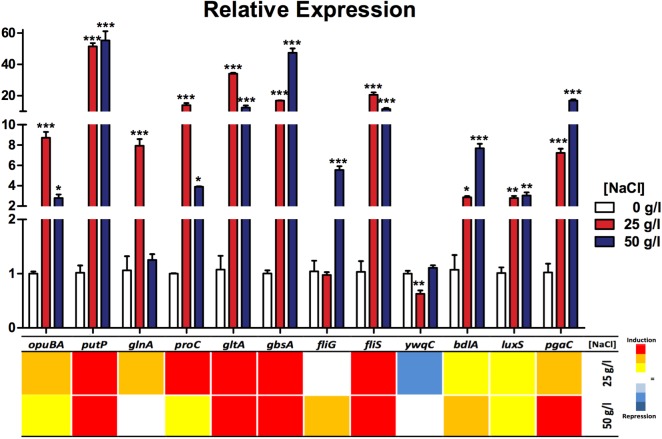
Relative expression of genes related to osmotolerance processes in *Exiguobacterium* sp. SH31 under different NaCl conditions. Data represent an average of three independent experiments with three technical replicates each (^∗^*p* < 0.05; ^∗∗^*p* < 0.01; ^∗∗∗^*p* < 0.001).

To correlate the gene expression with the phenotypic experiments and gain insight on the participation of different processes that lead (as a whole) to the osmotolerance capacity of this strain, we measured the transcript of several genes of interest that participate in processes like transport systems, compatible solutes biosynthesis and resistance to stress conditions as motility and biofilm production. Gene expression (quantified by qRT-PCR) shows a clear induction in all measured genes in response to the salinity conditions (25 and 50 g/l), compared to the levels found in 0 g/l NaCl (**Figure 6** and **Supplementary Table S6**). By functional grouping, we observed that those genes related to transport (*opuBA, putP*) were up to 50-fold in expression as were genes related to compatible solutes (*proC, glnA, gltA, gbsA*). Furthermore, expression of genes related to motility (*fliS, fliG*), biofilm, and stress response (*bdlA, pgaC, luxS*) were also induced, but not as high as the previous groups, except for the *ywqC* gene (biofilm formation) which was the only one that showed repression under the 25 g/l NaCl condition and no difference in the 50 g/l NaCl condition, with respect to controls.

## Discussion

Previous studies have shown there is a high presence of *Proteobacteria*, and also the presence of *Firmicutes* (but in lower abundance), in Salar de Huasco (Dorador et al., 2013; Aguilar et al., 2016; Molina et al., 2016). Regarding cultivable-dependent techniques, most studies are focused on using selective media to cultivate groups of halophilic prokaryotes and primarily focus in *Archaea* or extreme halophilic bacteria because of the biochemical strategies used to survive in high salt concentration. These halophilic bacteria are present in our study site (**Table 1**). In this context, members of Gammaproteobacteria (i.e., *Halomonas* and *Salicola*) and *Firmicutes* (*Bacillus*) are the most recurrent isolates identified from cultivable diversity studies in saline systems (Rohban et al., 2009; Sabet et al., 2009; Vahed et al., 2011; Luque et al., 2014; Kalwasinska et al., 2017). Strains with different grades of salt tolerance that belong to *Pseudomonas, Pseudoalteromonas*, and *Staphylococcus* genera have been isolated from two high altitude Andean lakes in Argentina (Flores et al., 2009). All these groups, besides members of *Idiomarina, Marinobacter*, and *Salinibrivio* genera, have been isolated from a saline lake in Romania, and its halotolerance capabilities have also been determined (Crognale et al., 2013). Strains of *Shewanella* and *Aeromonas* genera have been isolated from Salar de Aguas Caliente and research was focused on its capabilities of resistance to UV radiation (Demergasso et al., 2010). The effect of several stress factors were studied in an *Enterobacter* strain isolated from a high-altitude Andean saline lake (Dib et al., 2008). One member of the *Vibrio* genus, *Vibrio ruber*, is one the most studied halophilic eubacteria (Ventosa et al., 1998), and members of this genus have also been found in non-marine systems such as Salar de Atacama, where the relation between pigmentation and salt tolerance was investigated (Gallardo et al., 2016).

The *Exiguobacterium* genus has been widely described as a highly diverse group that are found in different habitats, which include extreme environments (Vishnivetskaya et al., 2009; Kasana and Pandey, 2018). However, bacteria in this genus are not reported as common in saline environments (Oren, 2015; Ventosa et al., 2015), several *Exiguobacterium* strains have been isolated from saline lakes with the aim of studying their responses to different stress factors, such as UV radiation and heavy metals (Flores et al., 2009; Ordoñez et al., 2009, 2013; Demergasso et al., 2010; Castro-Severyn et al., 2017; Strahsburger et al., 2017). Interestingly, results regarding the phylogenetic analysis of the phylotype represented by strain SH31 (**Supplementary Figure S1**) show similarity with the *Exiguobacterium* strain, AC-CS-C2 isolated from Salar de Aguas Calientes, Chilean Altiplano (Demergasso et al., 2010). However, our group recently revealed that the *Exiguobacterium* strain, S17, was the most similar isolate to strain SH31 using ANI analysis with available *Exiguobacterium* genomes (Castro-Severyn et al., 2017). Strain S17 was isolated from the Argentinian Altiplano (Lake Socompa), which had similar environment conditions to Salar de Huasco (Ordoñez et al., 2013). Moreover, *Exiguobacterium* strains that were isolated in this work have ranges of tolerance to NaCl between 0–50 g/l (**Table 2**), which is the highest described to date. It is important to note that the response to salt has not been previously studied in this group of microorganisms, only salt tolerance values have been mostly reported in type-strains description studies (**Supplementary Table S1**). In this way, these recurrent *Exiguobacterium* strains are candidates to relate saline adaptations with the viability of growth in a wide range of salt concentrations, analogous to the salinity values measured in Salar de Huasco.

The morphology shown by the SH31 strain corresponds with the one described in Collins et al. (1983), for this genus. However, it has been stated that the *Exiguobacterium* genus has morphological diversity (ovoid, rods, double rods, and chains), which depends on the species, strain, and environmental conditions (Vishnivetskaya et al., 2007, 2009). The strain SH31 presents a polar flagellum (**Figure 1**), which has also been observed in *E. marinum* that is isolated from the Yellow Sea in Korea (Kim et al., 2005). On the contrary, most of the described strains-types present with peritrichous flagellation; for instance, *E. auriantiacum, Exiguobacterium undae, Exiguobacterium antarcticum*, and *Exiguobacterium aestuarii* (Collins et al., 1983; Frühling et al., 2002; Kim et al., 2005). Despite the flagellation type, all the described strains of the *Exiguobacterium* genus are classified as motile (e.g., Collins et al., 1983; Frühling et al., 2002; López-Cortés et al., 2006), but the effect of salinity in the presence of flagella and motility have not been previously studied in these microorganisms. In addition, the versatile *Exiguobacterium* genus is a pigmented group of Gram-positive bacteria (Kasana and Pandey, 2018), and strain SH31 is not the exception, presenting with orange colonies (**Supplementary Figure S2A**) and showing a peak maximum absorbance in the visible wavelength region, between 400 and 500 nm (**Supplementary Figure S2B**), which is a typical pattern of absorption spectrum of a carotenoid (Liakopoulou-Kyriakides and Kyriakidis, 2002). Moreover, several members of this genus, which include *E. auriantiacum, E. undae, E. antarcticum, Exiguobacterium sibiricum, E. aestuarii*, and *E. marinum*, have shown the presence of orange pigmentation that have different intensities (Collins et al., 1983; Frühling et al., 2002; Kim et al., 2005; Rodrigues et al., 2006).

Carotenoid biosynthesis in microbes is a well-regulated mechanism that is dependent on the environmental conditions and stress (Bhosale, 2004). The enzymes phytoene synthase (*crtB*), phytoene desaturase (*crtI*), and lycopene cyclase (*crtY*) are essential in the carotenoid biosynthetic pathway, specifically responsible for the biosynthesis of both acyclic and cyclic carotenoids (Vachali et al., 2012). Carotenoid synthases appear to play the role of pathway gatekeeper; however, enzymes that function downstream in the pathway are less specific and appear to recognize only a particular motif of the substrate (Umeno et al., 2005). In the case of the strain SH31, the synthesis of acyclopenoic lycopene is probable due to the presence of phytoene synthase (*crtB*), and two desaturase enzymes (*carC* and *crtD*; **Supplementary Figure S2C**). The four steps of conversion of phytoene to lycopene have been demonstrated by the cooperation of two desaturases enzymes in the gram-negative bacterium, *Myxococcus xanthus* (Iniesta et al., 2007). In many organisms, carotenoids act as an antioxidant by neutralizing free radicals and thereby prevent oxidative damage to the cells (Köcher et al., 2009; Vachali et al., 2012). Furthermore, transcriptional profiles associate with general stress response in *B. subtilis* and revealed the expression of genes with a potential protective function. These include *yisP*, which is similar to carotenoid synthases (Price et al., 2001). Pigment biosynthesis has been extensively studied because of its importance in healthcare and food industries (Vachali et al., 2012).

Despite that the genus *Exiguobacterium* is prevalent and adapted to various environments (from cold environments to hot springs), most of these studies have focused on describing new strains and investigating their ability to grow over a wide range of temperatures (Vishnivetskaya et al., 2009; Kasana and Pandey, 2018). Tolerance to salt has been determined (in a basic way) in some members of the genus *Exiguobacterium*, described as type-strains (**Supplementary Table S1**). Although several strains of the genus *Exiguobacterium* have been isolated from saline environments, only the work of Rebollar et al. (2012) focused on studying halophilic properties to explore the influence of ecological factors on the evolution of bacterial populations. To date, strain SH31 shows the highest tolerance to salt that is described for this genus (**Figure 2** and **Supplementary Table S1**); therefore, it is an interesting candidate to study adaptation mechanisms against a wide range of salinities.

As mentioned before, the presence of flagellum (polar and peritrichous flagellation) and motility have been widely described in *Exiguobacterium* strains (Vishnivetskaya et al., 2009), but our results are the first that relate salinity with the presence of flagellum and motility in this genus (**Figure 3**). A negative effect of high salinity (∼70 g/l NaCl) on motility (Steil et al., 2003), and a repression of genes involved in chemotaxis and motility were observed in the Gram-positive *Bacillus subtilis* by using proteomic and transcriptomic approaches (Hoffmann et al., 2002; Steil et al., 2003). Conversely, the motility of the moderately halophilic Gram-positive *Halolactibacillus halophilus* is strictly dependent of chloride, and an increase in chloride concentration led to a simultaneous increase in motility. Moreover, cells grown in the absence of chloride were lacking flagella; however, it was restored upon the addition of chloride (Roeßler et al., 2000). A recent report indicates that the salt concentration is dependent on motility in a pigment halotolerant *Vibrio* strain isolated from Salar de Atacama, but motility was inhibited in higher salt concentrations (150 g/l NaCl; Gallardo et al., 2016). Substantial energy is needed for flagellar biosynthesis and bacteria are able to inhibit certain processes in response to stress, motility was reduced, and biofilm formation was seen in SH31 (**Figure 4**) a general bacterial response to osmotic stress found in diverse organisms, as it was shown in *Pseudomonas putida* (Bojanovič et al., 2017). Some factors that induce biofilm maturation, including in *Pseudomonas* and *Shewanella*, are changes in oxygen or carbon substrate concentration, pH, or other chemical parameters (Gjermansen et al., 2005).

Salt-dependent changes in the cell membranes are reflected in the types of phospholipids that dominate it and the types of fatty acid chains present in these lipids. Since the fatty acid composition is also influenced by temperature, it can be expected a complex interrelation between salinity and temperature (Ventosa et al., 1998). Even though changes in the fatty acid pattern were not observed in strain SH31, due to the presence of salts (**Table 3**), the branched-chain fatty acids, such as 15:0, are dominant in some halophilic Gram-positive bacteria (Monteoliva-Sanchez et al., 1989). Moreover, the concentration of these branched-chain fatty acids variates with salinity (Russell, 1993). On the other hand, the amount of shorter chains increased and the presence of chains that were unsaturated were observed in *H. halophilus* after increases the salinity in the culture medium. These changes might compensate for the increase in the arranging and rigidity of the phospholipid and sulfoglycolipid polar heads in high-salt environment, as a consequence contributes to the homeostasis of membrane fluidity and permeability in salt stress conditions (Lopalco et al., 2013). The remarkable differences between the SH31 strain and other members of *Exiguobacterium* genus support the high degree of variability at genome level between strains of the genus recently demonstrated; additionally, it was found that SH31 clusters with the S17 strain that was isolated from an environment with similar poly-extremophile environment (Castro-Severyn et al., 2017).

Genomic analysis shows that some type of genetic redundancy related to osmotolerance functions may increase the ability of some strains (*E. marinum* DSM 16307, *Exiguobacterium enclense* NIO-1109, *E. undae* 190-11) to tolerate higher concentrations of salts in their environment. This stems from the identification of a positive correlation between the genomes that have the largest number of copies of some genes (*opuCA* and *gltR*) and their reported resistance (**Figure 5** and **Supplementary Table S1**; Rodrigues et al., 2006; Dastager et al., 2015).

In almost every process evaluated, the relative gene expression was induced by the presence of the NaCl concentrations: 25 and 50 g/l (**Figure 6**). Genes were separated into functional groups; those related to transport systems (*opuBA* and *putP*) were induced in both conditions (25 and 50 g/l NaCl) compared to the control. *putP* was, in particular, strongly overexpressed. This gene product is a high-affinity proline/sodium symporter, which carries out the uptake of extracellular proline, which can be used as a source of nitrogen and carbon (Moses et al., 2012); on the other hand, *opuBA* is part of a choline transport mechanisms commonly related to cell osmoprotection (Kappes et al., 1999) and this gene is also induced, but in a smaller amount. Also, it seems that for this gene, the condition of 25 g/l caused a greater effect on its expression compared to the 50 g/l condition. Choline, a compatible solute transported by the product of this gene and has been proven to be directly related to the ability of several bacteria to resist NaCl (Sand et al., 2014; Scholz et al., 2016).

The expression of all measured genes related to compatible solute biosynthesis turned out to occur in both salt conditions. We selected a representative gene of several compounds biosynthesis, like proline (*proC*), glutamate (*gltA*), betaine (*gbsA*), and glutamine (*glnA). glnA* was the only that did not show a significant increase related to the control condition, and it was only observed in the 50 g/l condition. These compatible solute strategies allowed the bacteria to cope with high salinity environments and were widely studied in several organisms that thrive under adverse conditions (Kempf and Bremer, 1998; Santos and Da Costa, 2002).

Motility has been reported as a strategy used by different types of bacteria to cope with adverse conditions (Li et al., 1993). Our results reveal a significate increase in flagellar assembly chaperon gene *fliS* in both NaCl conditions, which is expected because the product of this gene is necessary during flagella biosynthesis because it binds to the most abundant protein of the flagellum (FliC) to facilitate its export to the filament in formation (Auvray et al., 2001; Muskotál et al., 2006). Also, *fliC* gene expression has been reported as induced by the presence of chloride ions (Roeßler and Muller, 2002). Corresponding to these results, in the presence of 4% NaCl, *Tistlia consotensis* cellular proteome showed that flagellin was being upregulated and was one of the most abundant proteins in the exoproteome (Rubiano-Labrador et al., 2015). Our results reveal that *fliG* gene expression is significantly increased in the 50 g/l condition of NaCl, which may be due to the fact that this gene product is less required than FliC or FliS during flagella biosynthesis (Terashima et al., 2008).

It should be noted that these results do not correspond to those observed in plate motility experiments, in which this capacity was totally inhibited at 50 g/l of NaCl; this result may reflect some type of post-transcriptional regulation (Chatterjee et al., 2010). This phenomenon has been previously associated as a response to a variety of stress conditions, specially closely related to biofilm formation as a protection structure in which the bacteria tend to seek each other and remain embedded in the substrate (Mitchell and Kogure, 2006; Mika and Hengge, 2013). Another reason for this phenomenon could be that flagella transcription is induced by environmental signals as was observed in bacteria grown in 25 and 50 g/l NaCl; however, biofilm formation was most induced at 50 g/l NaCl this could prevent the bacteria from moving, in turn increase resistance and saving energy.

Finally, genes related to biofilm (*pgaC*), cell chemotaxis (*bdlA*), and signaling (*luxS*) were induced in the NaCl conditions; on the contrary, *ywqC* or *tkm* (capsule/biofilm) was found to be significantly decreased in the 25 g/l condition and was not changed in 50 g/l of NaCl. It has been found that many of the reactions related to biofilm formation are upregulated in response to salinity (Bojanovič et al., 2017). LuxS is a signaling protein that mediates quorum sensing between certain species, as well as biofilm formation and motility. Also, its expression has been reported as salt dependant in *H. halophilus* (Sewald et al., 2007; Hardie and Heurlier, 2008). Biofilm is a resistance strategy used by many different bacteria to cope against environmental change or pressures (Hall-Stoodley et al., 2004; Poole, 2012); this correlates with our findings of significant increases in *pgaC* and *bdlA* gene expression, which participate in biofilm synthesis and dispersion, respectively (Wang et al., 2004; Morgan et al., 2006). YwqC (TkmA) is a tyrosine-kinase modulator that interacts cognately with PtkA allowing it to phosphorylate its target proteins in post-translational regulation during biofilm formation (Jers et al., 2010; Gao et al., 2015). This was the only gene that showed decreased expression in the under 25 g/l NaCl condition.

## Conclusion

In sum, our results show that isolated Phylotypes include strict halophiles and halotolerants, of which *Exiguobacterium* is a recurrent halotolerant and cultivable genus from Salar de Huasco that was able to tolerate up to 50 g/l NaCl (the highest reported one to date for this genus), showing its best growth behavior at 25 g/l as well as its highest motility. Furthermore, it seems that the fatty acid composition is not altered by salinity; furthermore, biofilm formation is affected, which has been widely reported as a strategy of resistance to environmental pressures. Additionally, we have found that gene expression results are in absolute coherence with phenotypic results and genomic information in regards to the resistance capacity. We can conclude that the stress conditions, caused by the salt on the cell, effectively induce the expression of genes related to several processes of adaptation to ensure survival. Moreover, the wide set of strategies presented and used by this strain to thrive under environmental stress conditions predict that it would be a suitable model for evolutionary adaptation studies.

## Author Contributions

FR, JL, SB, and KH performed field work and processed samples. CPS and FR conceived and designed the study. FR, JC-S, CS, PA, JF, and CP-E performed the experiments. JC-S, SB, PA, CA, and FR analyzed the data. FR, CA, BF, DA, and CPS contributed with reagents, materials, and analysis tools. JC-S, CP-E, FR, and CPS wrote the paper. All authors read and approved the final manuscript.

## Conflict of Interest Statement

The authors declare that the research was conducted in the absence of any commercial or financial relationships that could be construed as a potential conflict of interest.

## Footnotes

1 www.graphpad.com
