# Supplementary figures and images for: Characterization and Salt Response in Recurrent Halotolerant Exiguobacterium sp. SH31 Isolated From Sediments of Salar de Huasco, Chilean Altiplano
Source: Front Microbiol. 2018 Sep 20;9:2228. doi: 10.3389/fmicb.2018.02228 (PMC6158405; doi:10.3389/fmicb.2018.02228)

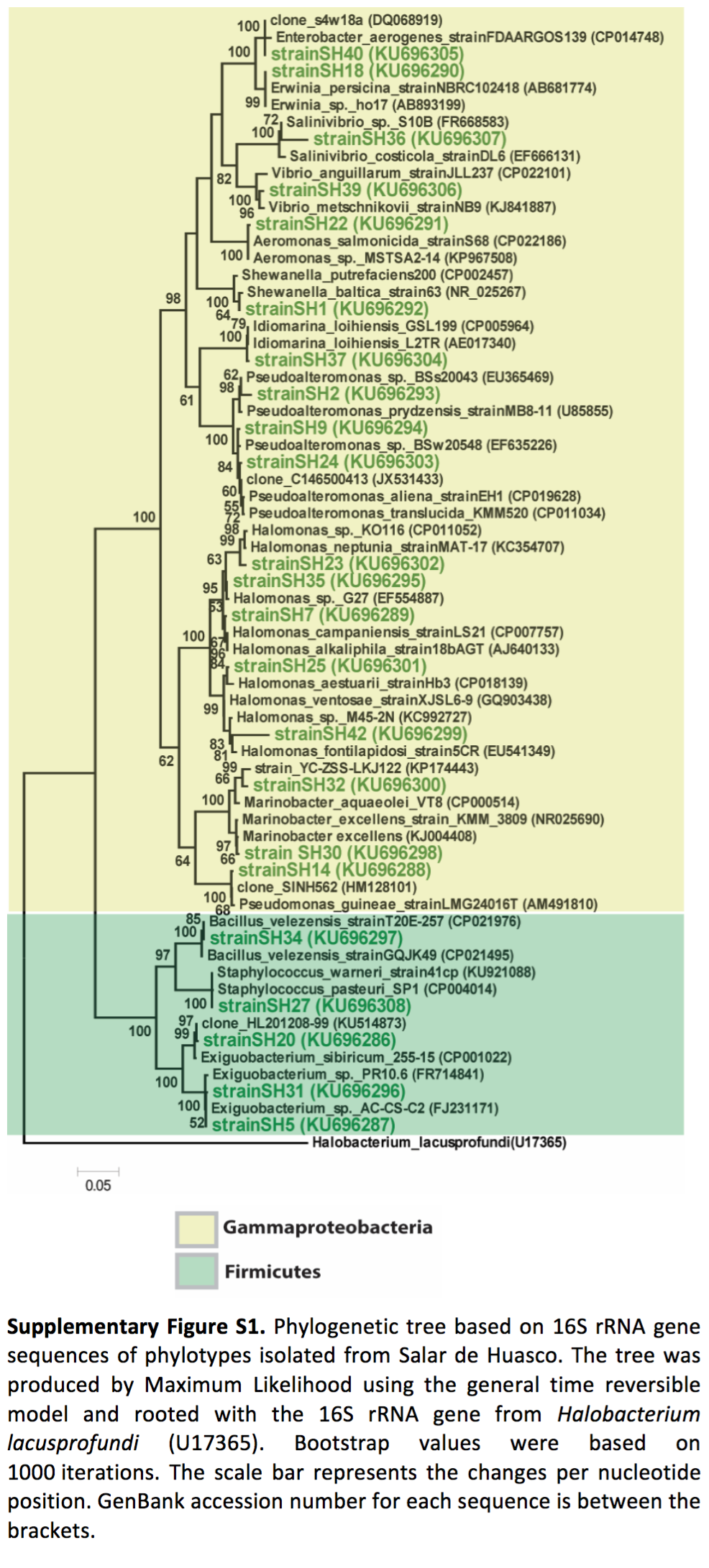

Supplement: Supplementary file 1 [file Image_1.PNG]

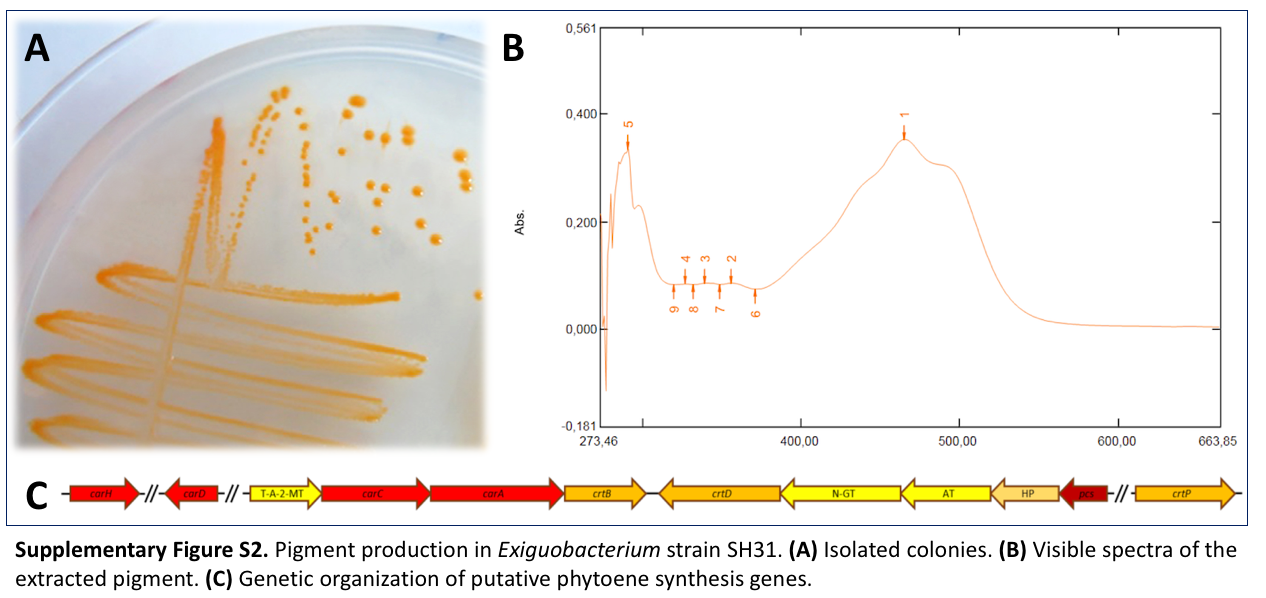

Supplement: Supplementary file 2 [file Image_2.JPEG]
